# Beyond the Scale of Big Data

**DOI:** 10.3389/fdata.2018.00001

**Published:** 2018-07-10

**Authors:** Huan Liu

**Affiliations:** Computer Science and Engineering, Arizona State University, Tempe, AZ, United States

**Keywords:** big data, data mining, privacy, security, trust, integration

Voluminous data brings out a new heat-wave of machine learning with the concomitant escalation of computing power. People of all walks of life have witnessed unprecedented achievements by tapping on data. Complementing the adage of “knowledge is power,” we learn that “data is the new oil.” The pervasive big data has demonstrated its great potential in artificial intelligent (AI) research and advancement. We are getting better at discovering knowledge from data and acquiring intelligence from information. Data, as an indispensable source for data mining and machine learning, can only become bigger and more. Big data also exhibits characteristics that go beyond scale. Big data is multifaceted including disparate dimensions such as social, spatial, relational, educational, structured, unstructured or semi-structured, and user-generated. Big data evolves in temporal or streaming forms. With such a big variety of data, big data is not just confined in centralized database management systems or warehouses as in the recent past. Big data can be produced anytime anywhere, shared explicitly or implicitly, linked automatically, and obtained by easy-to-use tools via crawling or scraping.

All these new developments of data production result in some obvious questions for and/or from researchers, practitioners, and concerned citizens. Two prominent issues at the heart of this specialty section of “Data Mining and Management” are *privacy* and *integration* of data mining and data management in the era of big data. The issue of privacy cannot be overstated given recent egregious large-scale privacy breaches and the promulgation of the General Data Protection Regulation (GDPR), a newly taking-effect European regulation.

Privacy encompasses topics ranging from data, individuals/data producers, consumers, and analyzers to owners. It is clear that all stakeholders of data have their unique concerns. First of all, useful data can often be personal data: for example, think about healthcare data and e-medical records, when different patients of rare diseases can share their data, the data potency emerges to fight against the odds in terms of differed treatments and effects; but in the meantime, every patient has their right of privacy in order to keep a private matter private. Another example of privacy is about individuals who generate data while making Web search. With information overloading, search engines come to the rescue. Online users contribute their data during their search and the collective data makes search engines so powerful that one can find the needed information on the fly. The more one searches, the more traces they leave behind. As a result, users' search histories become unique signatures that can be used to unmistakably identify themselves. Now we encounter a *search paradox* in the context of privacy, i.e., we rely on “search” to help us find the information we need quickly (a.k.a., utility), but our search also undoubtedly reveals our identity, and thus signifies the loss of our privacy. Maximum privacy entails minimum search which renders us helpless when facing information overloading. A challenge for researchers is whether it is possible for users to retain utility with little loss of privacy.

Two closely related issues are data *security* and public *trust*. Secure data platforms strengthen data security which in turn helps boost public trust. With established trust and guaranteed privacy, users shall be more willing to share their data and shared data can become more relevant and beneficial. Another associated issue in dealing with data is the one of *ethics*. Ultimately, the stakeholders of data are humans. As new capabilities are being developed, ethics evolve. Therefore, research on this front is also urgently needed. Security, trust, and ethics are all important topics this specialty section of DMM is hoping to see more activity through various forms of publications.

Seamless integration of data mining and data management is more pressing than ever in the era of big data. Data management has a glorious, long history and has helped build reliable and working systems that make possible a plethora of data-centric applications from pervasive banking systems to current cloud computing. Data mining is a relative young field that became flourishing due to the accumulation and availability of data and the need for finding actionable patterns from the treasure trove of data. Traditionally, the two fields develop on their own paces, answering their endogenous challenges, in a sense, independently, with sporadic cross-over in the past, but their merging trend seems natural and even becomes necessary facing privacy and security challenges. All the exciting news of big data and its triumphs may give us the illusion that all problems are solved and we are given some turn-key solutions to enable data to work hard for us. The reality is that though data in each silo is not small, it is still largely segmented due to many concerns; not least of which, as mentioned, are privacy and security. For example, all businesses would like to maximize the value of their data, but no business could simply share their data. Without data sharing, the value of data cannot be fully realized; with data sharing, privacy and security can be at risk. One of the feasible solutions to *this data-sharing paradox* is to integrate data mining with data management; when they are integrated seamlessly, the privacy and security concerns can be significantly mitigated. It's always easier said than done. The integration has been attempted before. The need for integration becomes imperative lately due to increasing computing power and accessible infrastructures such as cloud services. This Specialty Section of Data Mining and Management provides a conducive and interdisciplinary platform that fosters scholarly conversation between research communities and industry, and it enables fruitful collaborations and the exchange of high-quality research outputs. Being open-minded, inclusive, innovative, and Open Access based, we aim to cover a wide range of topics within this framework, from privacy-preserving data sharing and scalable data mining to intelligent data management.

## Author contributions

The author confirms being the sole contributor of this work and approved it for publication.

### Conflict of interest statement

The author declares that the research was conducted in the absence of any commercial or financial relationships that could be construed as a potential conflict of interest.

